# Evaluation of large language models for VI-RADS reports: a comparative analysis of zero-shot and few-shot prompting

**DOI:** 10.1186/s12880-026-02334-y

**Published:** 2026-04-11

**Authors:** Ahmet Halis, Deniz Celiker

**Affiliations:** 1 Department of Urology, Yedikule Chest Diseases and Thoracic Surgery Training and Research Hospital, Kazlıçeşme Neighborhood, Belgrat Kapı Yolu Street No:1, Istanbul, Turkey; 2 Department of Radiology, Acıbadem University Atasehir Hospital, Istanbul, Turkey

**Keywords:** Bladder cancer, VI-RADS, Large language models (LLMs), Few-shot learning, Artificial intelligence in radiology

## Abstract

**Introduction:**

Accurate preoperative staging is vital in bladder cancer management, particularly for assessing muscle invasion. Multiparametric MRI (mpMRI) combined with the Vesical Imaging–Reporting and Data System (VI-RADS) offers a non-invasive and standardized approach for tumor stratification. However, inter-observer variability among radiologists remains a significant limitation. This study investigates the performance of three large language models (LLMs)—ChatGPT (OpenAI; GPT-5.2), Gemini (Google; Gemini 2.0), and Copilot (Microsoft; Copilot based on GPT-5 architecture)—in classifying bladder lesions according to VI-RADS using zero-shot and few-shot prompting strategies.

**Materials and Methods:**

A synthetic dataset of 100 simulated bladder cancer cases was developed from expert-crafted synthetic radiology reports based on structured text descriptors and expert consensus, comprising 20 cases for each VI-RADS category (1–5). Each case included structured imaging descriptors aligned with VI-RADS scoring criteria. The LLMs were evaluated under zero-shot (no examples provided) and few-shot (with illustrative examples) prompting conditions. Performance metrics included accuracy, macro F1 scores, and Cohen’s kappa, with statistical significance assessed using McNemar’s test.

**Results:**

Few-shot prompting significantly improved the classification performance of ChatGPT (OpenAI; GPT-5.2) (accuracy: 94%, F1: 0.939) and Gemini (Google; Gemini 2.0) (accuracy: 83%, F1: 0.823) compared to zero-shot. In contrast, Copilot (Microsoft; Copilot based on GPT-5 architecture)‘s accuracy (53%) and F1 score (0.503) declined under few-shot conditions. ChatGPT (OpenAI; GPT-5.2) demonstrated the highest consistency in identifying high-risk lesions (VI-RADS 4–5), followed by Gemini (Google; Gemini 2.0).

**Conclusion:**

Few-shot prompting enhances LLM performance in VI-RADS classification, particularly for ChatGPT (OpenAI; GPT-5.2) and Gemini (Google; Gemini 2.0). These findings highlight the potential of AI tools to support radiological decision-making in bladder cancer staging. Further studies using real imaging data and explainable AI are warranted.

**Supplementary Information:**

The online version contains supplementary material available at 10.1186/s12880-026-02334-y.

## Introduction

Bladder cancer, known for its high incidence, significant treatment expenses, and considerable morbidity and mortality rates, highlights the essential need for early detection, precise staging, and effective treatment planning [1]. The staging of tumors, especially the T stage, is crucial in determining the treatment course for bladder cancer [2].

Accurate staging of bladder cancer requires a thorough assessment of clinical, radiological, and histopathological findings. Inadequate data collection, especially in determining muscle invasion during transurethral surgery, may lead to delayed diagnoses and potential disease progression. As a result, the importance of precise preoperative staging is becoming increasingly critical [3].

Multiparametric magnetic resonance imaging (mp-MRI) is progressively becoming the preferred imaging modality over CT for bladder cancer due to its enhanced spatial resolution and improved ability to accurately delineate the bladder layers and surrounding anatomical structures. Additionally, mp-MRI has demonstrated high diagnostic accuracy in distinguishing NMIBC from MIBC and in assessing the likelihood of extravesical extension [4]. The Vesical Imaging–Reporting and Data System (VI-RADS), established in 2018 through expert consensus, standardized the acquisition, interpretation, and reporting of mp-MRI for bladder cancer [5]. In radiological practice, VI-RADS scoring is typically performed using standard MRI sequences, including T2-weighted and post-contrast T1-weighted images for anatomical assessment, as well as diffusion-weighted imaging (DWI) and dynamic contrast-enhanced MRI (DCE-MRI) for evaluating physiological characteristics [6].

Large language models (LLMs) have demonstrated significant capabilities in interpreting and processing complex medical data, including text-based radiological reports [7]. The integration of AI-driven models in radiological assessment presents an opportunity to enhance diagnostic consistency and reduce inter-observer variability [8].

One such area where AI assistance could be beneficial is the Vesical Imaging-Reporting and Data System (VI-RADS), which is used to standardize the assessment of bladder cancer based on multiparametric magnetic resonance imaging (mpMRI). VI-RADS plays a crucial role in determining the likelihood of muscle invasion, guiding clinical decision-making for bladder cancer management. However, variability in radiologists’ interpretations can affect diagnostic accuracy and subsequent treatment decisions.

This study aims to evaluate the performance of three LLMs—ChatGPT (OpenAI; GPT-5.2), Gemini (Google; Gemini 2.0), and Copilot (Microsoft; Copilot based on GPT-5 architecture), in classifying bladder lesions according to the VI-RADS criteria. Specifically, we will assess the accuracy of these models under different prompting techniques, including zero-shot and few-shot learning to enhance contextual understanding. Our hypothesis is that optimized prompting strategies will improve the accuracy of LLMs in assigning VI-RADS scores, particularly in borderline cases where radiological interpretation is more challenging. By systematically comparing the performance of these AI models, this study seeks to explore their potential integration into radiology workflows as an AI-assisted decision-support system for VI-RADS classification.

## Materials and methods

**Fig. 1:**
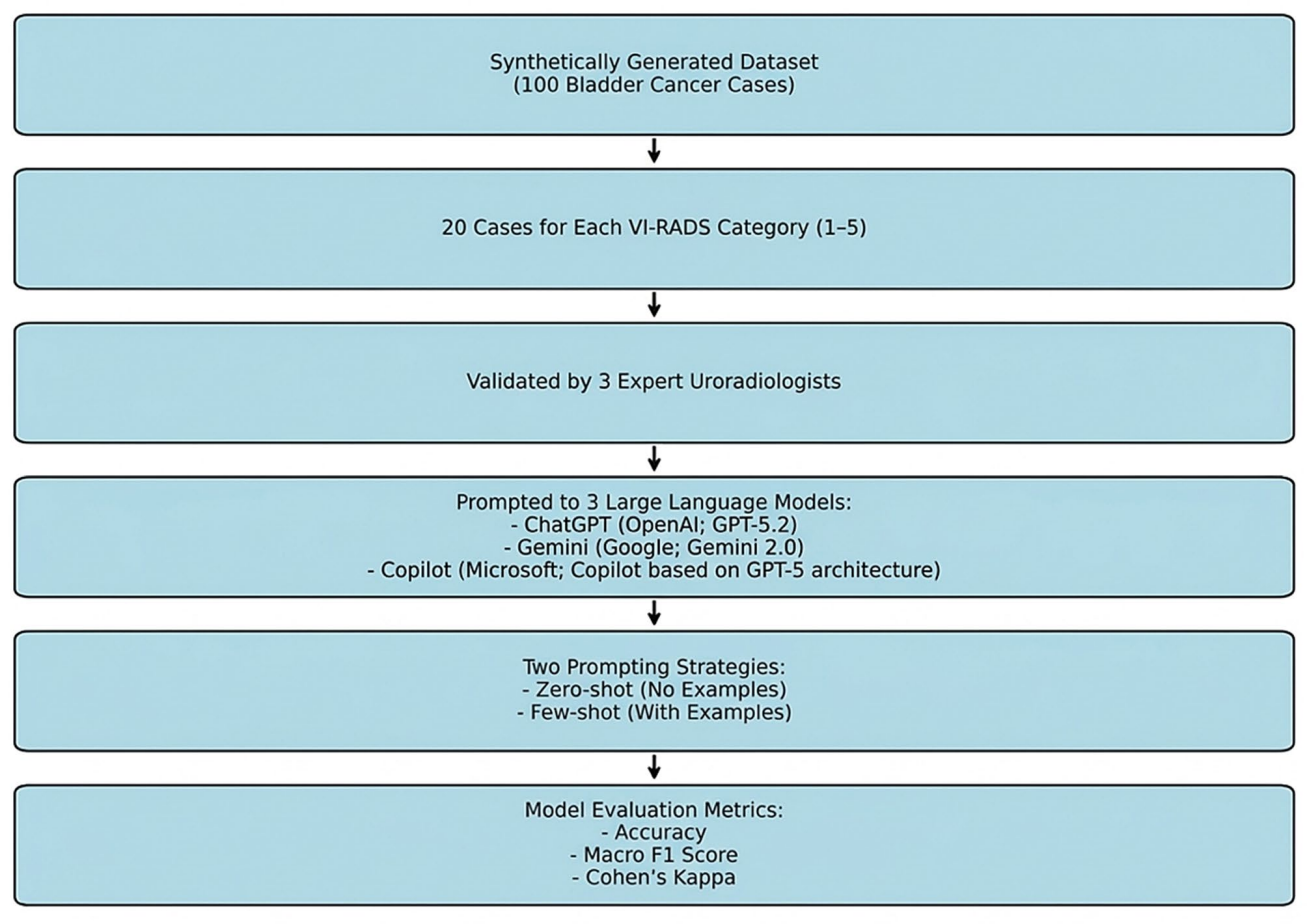
Study Flowchart. Flowchart illustrating the overall study design. A total of 100 synthetic bladder cancer cases were created, with 20 cases allocated to each VI-RADS score group (1–5). The dataset was validated by three expert uroradiologists for clinical consistency. Subsequently, three LLMs— ChatGPT (OpenAI; GPT–5.2), Gemini (Google; Gemini 2.0), and Copilot (Microsoft; Copilot based on GPT-5 architecture)—were prompted to classify the cases under two distinct prompting strategies: zero-shot (no examples provided) and few-shot (with representative examples). Model performance was evaluated using accuracy, macro F1 score, and Cohen’s kappa coefficient

A synthetic dataset of 100 bladder cancer cases was developed to simulate real-world clinical presentations of bladder tumors, with 20 cases representing each VI-RADS score category (1–5), resulting in a total of 100 distinct clinical scenarios. This dataset was designed to ensure high clinical validity while maintaining full control over data variability. The structured dataset incorporated key radiological features relevant to the Vesical Imaging-Reporting and Data System (VI-RADS) classification, including: Tumor morphology (size, shape, wall thickness). Bladder wall layer involvement (muscularis propria invasion assessment). Diffusion characteristics (DWI signal intensity, apparent diffusion coefficient). Contrast enhancement patterns (non-enhancing, minimal, or measurable enhancement on DCE MRI). Since the dataset was synthetically generated, it does not include actual patient data. Instead, it was constructed using predefined imaging and clinical parameters derived from established medical literature and expert consensus to reflect real-world bladder cancer cases. The synthetic approach allowed the inclusion of a diverse range of tumor presentations while ensuring standardization across cases. Each report in the dataset was designed to be representative of real-world cases and was validated based on the consensus of three experienced uroradiologists to ensure consistency with clinical scenarios (See Fig. 1). The synthetic nature of the dataset eliminates concerns regarding patient confidentiality while enabling controlled analysis of VI-RADS classification performance. This study was reported in accordance with the TRIPOD-LLM reporting guideline. The completed TRIPOD-LLM checklist is provided as Supplementary Material.

## VI-RADS assessment and model evaluation

The VI-RADS scoring system was used to categorize bladder tumors based on the likelihood of muscle invasion. Each lesion was independently evaluated by a radiologist. The VI-RADS classification system assigns tumors into five categories:

VI-RADS 1–2: Low likelihood of muscle invasion.

VI-RADS 3: Indeterminate.

VI-RADS 4–5: High likelihood of muscle invasion.

Each case was assessed under different interpretative conditions:

**Zero-shot analysis:** The models were tested on the VI-RADS classification task without any prior exposure or training examples.

**Few-shot learning:** Models were provided with representative examples for each VI-RADS category to leverage prior contextual understanding and improve classification accuracy.

For the zero-shot setting, the models were provided with a single instruction requesting classification of the radiological report according to the VI-RADS system without any prior examples.

In the few-shot setting, representative examples of bladder MRI reports corresponding to each VI-RADS category (1–5) were provided before the target case to guide the classification task. The examples included structured textual descriptions of lesion morphology, diffusion characteristics, and enhancement patterns. In the few-shot prompting setting, ten representative examples were provided prior to the target case, including two examples for each VI-RADS category (scores 1–5). The complete prompt structure and example reports are provided in the Supplementary Material.

The LLMs evaluated in this study included ChatGPT (OpenAI; GPT-5.2), Gemini (Google; Gemini 2.0), and Copilot (Microsoft; Copilot based on GPT-5 architecture). All models were accessed in December 2025, and the latest publicly available versions at the time of access were used for all analyses. To ensure comparability across models, identical prompts and input data were used for each evaluation. All interactions were conducted through the publicly available web interfaces of the respective platforms using default inference settings. Each case was evaluated in a separate session to prevent contextual carryover between responses. Each model generated a single response for each case (single-pass inference). Temperature parameters, stochastic decoding strategies, and repeated inference with response aggregation were not modified or implemented, as interactions were performed through the publicly available web interfaces using default system settings. The exact prompts used for zero-shot and few-shot evaluations, along with representative examples, are provided in the Supplementary Material to facilitate transparency and reproducibility.

## Ethical considerations

Since this study was based entirely on synthetically generated clinical scenarios and did not involve any real patient data, institutional ethics committee approval and informed consent were not required.

### Statistical analysis

**Fig. 2:**
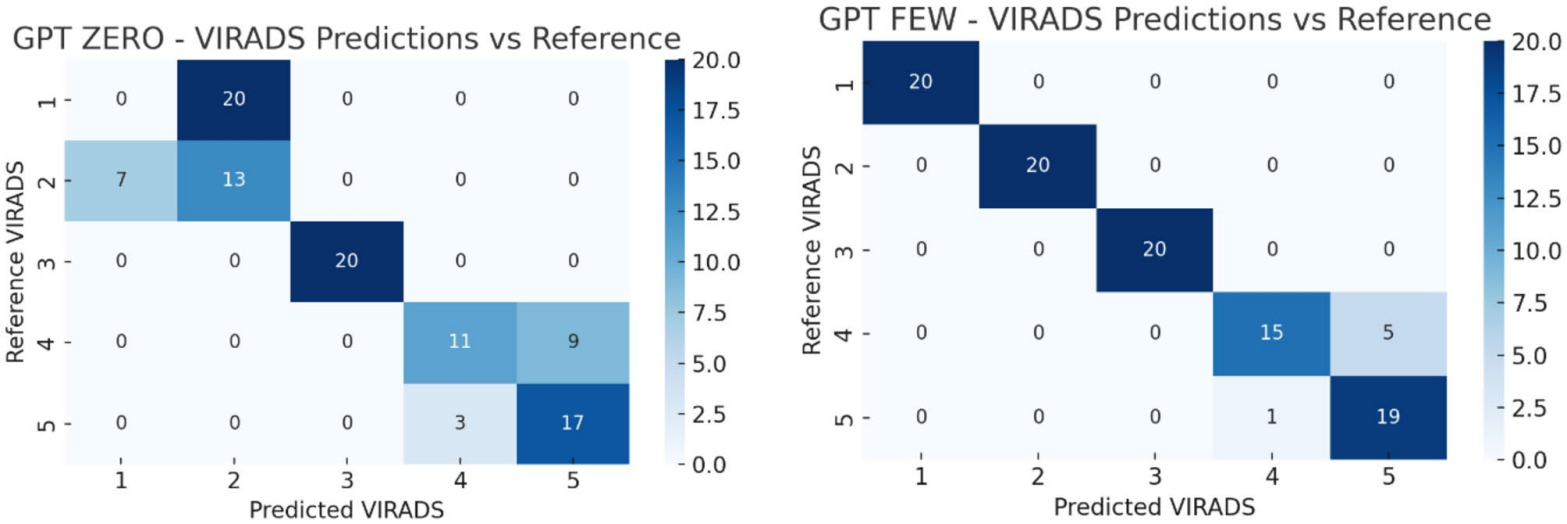
Distribution of ChatGPT (OpenAI; GPT-5.2) predictions versus reference VI-RADS scores

**Fig. 3:**
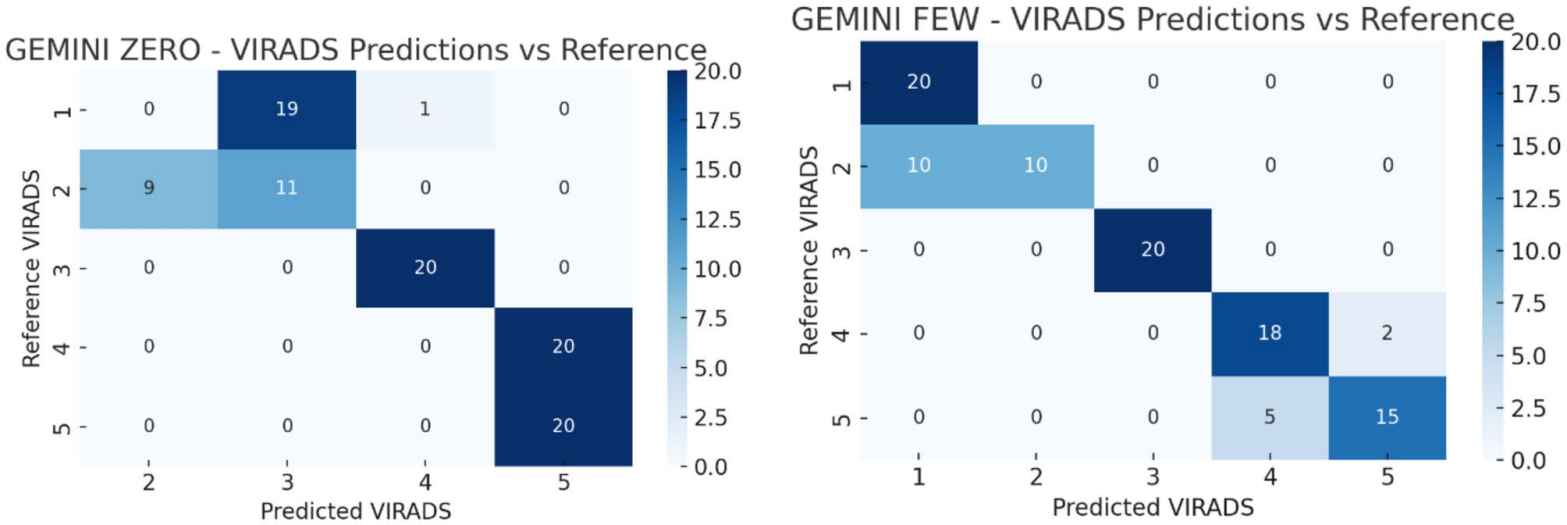
Distribution of Gemini (Google; Gemini 2.0) predictions versus reference VI-RADS scores

**Fig. 4:**
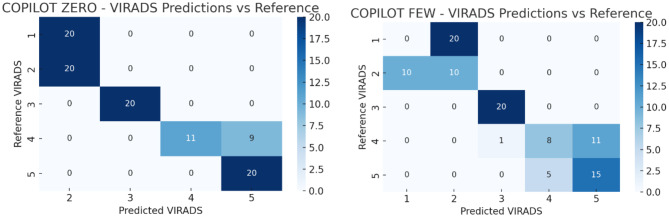
Distribution of Copilot (Microsoft; Copilot based on GPT-5 architecture) predictions versus reference VI-RADS scores. Fig. 2–4 demonstrate the prediction distributions of the three LLMs (ChatGPT (OpenAI; GPT-5.2), Gemini (Google; Gemini 2.0), and Copilot (Microsoft; Copilot based on GPT-5 architecture)) compared to the reference VI-RADS scores. In both ChatGPT (OpenAI; GPT-5.2) and Gemini (Google; Gemini 2.0), the few-shot setting resulted in better alignment with reference scores, especially for VI-RADS 3 to 5. Notably, ChatGPT (OpenAI; GPT-5.2) showed the most consistent predictions. Gemini (Google; Gemini 2.0) showed improvements in the few-shot setting, although misclassifications remained more common in lower categories. Copilot (Microsoft; Copilot based on GPT-5 architecture) exhibited inconsistent performance overall, with substantial deviation from reference scores, particularly in higher VI-RADS categories

All statistical analyses were conducted using Python (v3.9) and SPSS Statistics (v27.0, IBM Corp., Armonk, NY). Model performance was assessed by calculating accuracy, macro-averaged F1 scores, and Cohen’s kappa coefficients with respect to the reference VI-RADS classifications. Accuracy was defined as the proportion of exact matches between the AI-generated and reference scores. Macro F1 scores were computed to reflect the balance between precision and recall across all VI-RADS categories. Inter-rater agreement between each AI model and the reference was quantified using Cohen’s kappa (κ), interpreted as follows: poor (<0.20), fair (0.21–0.40), moderate (0.41–0.60), substantial (0.61–0.80), and almost perfect (0.81–1.00). Cohen’s kappa was selected because the agreement was evaluated between two raters at a time (the AI model and the reference classification). Multi-rater agreement statistics such as Fleiss’ kappa were not applied, as the models were not jointly evaluated as simultaneous raters but rather independently compared with the reference standard. To evaluate whether differences in accuracy between model versions (e.g., zero-shot vs. few-shot) were statistically significant, McNemar’s test with exact *p*-value computation was applied. McNemar’s test is appropriate for paired nominal data and is commonly used to compare the performance of two classifiers evaluated on the same dataset. In this study, each model generated predictions for the same set of cases, creating paired outcomes (correct vs. incorrect classification) that satisfy the assumptions of the McNemar test. Pairwise comparisons were performed across AI models for both the full sample and the high-risk (VI-RADS 4–5) subgroup. While repeated applications of McNemar’s test may increase the risk of type I error, this issue was addressed by applying Bonferroni correction for multiple comparisons. A *p*-value of <0.05 was considered statistically significant. To control for potential inflation of type I error due to multiple pairwise comparisons, *p*-values obtained from McNemar’s tests were adjusted using the Bonferroni correction method. The adjusted significance threshold was determined according to the number of pairwise comparisons performed. Figures were generated to visualize agreement patterns using confusion matrices in heatmap format (See Figs. 2, 3, 4).

## Results

The performance metrics of each LLM in both zero-shot and few-shot settings for VI-RADS classification are presented in Table 1. While ChatGPT (OpenAI; GPT-5.2) and Gemini (Google; Gemini 2.0) showed substantial performance improvements with few-shot prompting, Copilot (Microsoft; Copilot based on GPT-5 architecture) demonstrated a decline in accuracy, macro F1 score, and kappa in the few-shot setting compared to its zero-shot performance. ChatGPT (OpenAI; GPT-5.2) achieved an accuracy of 0.61 and a macro F1 score of 0.575 in the zero-shot setting, which improved significantly to 0.94 and 0.939, respectively, in the few-shot setting **(p = 0.001)**. Similarly, Gemini (Google; Gemini 2.0) showed a marked improvement in accuracy (from 0.29 to 0.83) and macro F1 score (from 0.257 to 0.823) when transitioning from zero-shot to few-shot **(p = 0.001)**. Copilot (Microsoft; Copilot based on GPT-5 architecture), however, demonstrated a different trend. While its zero-shot accuracy (0.71) and macro F1 score (0.639) were relatively high, these values declined in the few-shot setting to 0.53 and 0.503, respectively **(p = 0.001)**, indicating performance deterioration with few-shot prompting.

**Table 1:** Performance metrics of all AI models (zero-shot and few-shot) for overall VI-RADS classification

	ChatGPT (OpenAI; GPT-5.2) zero shot	ChatGPT (OpenAI; GPT-5.2) few-shot	Gemini (Google; Gemini 2.0) zero shot	Gemini (Google; Gemini 2.0) few shot	Copilot (Microsoft; Copilot based on GPT-5 architecture) zero shot	Copilot (Microsoft; Copilot based on GPT-5 architecture) few shot
Accuracy	0.61 (0.51–0.70)	0.94 (0.87–0.98)	0.29 (0.20–0.39)	0.83 (0.74–0.90)	0.71 (0.61–0.80)	0.53 (0.43–0.63)
Macro F1 Score	0.575 (0.48–0.67)	0.939 (0.88–0.98)	0.257 (0.18–0.34)	0.823 (0.73–0.89)	0.639 (0.54–0.73)	0.503 (0.41–0.59)
Kappa	0.512 (0.41–0.61)	0.925 (0.86–0.97)	0.113 (0.04–0.19)	0.788 (0.69–0.87)	0.638 (0.53–0.74)	0.412 (0.31–0.52)

When comparing models under the zero-shot condition, ChatGPT (OpenAI; GPT-5.2) and Copilot (Microsoft; Copilot based on GPT-5 architecture) performed comparably (*p* = 0.135), while Gemini (Google; Gemini 2.0) lagged significantly behind both (**p = 0.001** vs. ChatGPT (OpenAI; GPT-5.2) and Copilot (Microsoft; Copilot based on GPT-5 architecture)). In the few-shot setting, ChatGPT (OpenAI; GPT-5.2) outperformed both Gemini (Google; Gemini 2.0) (*p* = 0.014) and Copilot (Microsoft; Copilot based on GPT-5 architecture) **(p = 0.001),** whereas Copilot (Microsoft; Copilot based on GPT-5 architecture) also significantly underperformed compared to Gemini (Google; Gemini 2.0) **(p = 0.001)**. Overall, the few-shot setting substantially enhanced the performance of ChatGPT (OpenAI; GPT-5.2) and Gemini (Google; Gemini 2.0), while unexpectedly impairing Copilot (Microsoft; Copilot based on GPT-5 architecture)‘s classification performance.

When the analysis was restricted to high-suspicion cases (VI-RADS 4–5), the diagnostic performance of the models showed varying trends (Table 2). In the ChatGPT (OpenAI; GPT-5.2) group, few-shot prompting modestly improved accuracy (from 0.700 to 0.850), macro F1 score (from 0.693 to 0.848), and kappa (from 0.400 to 0.700), although the difference did not reach statistical significance (*p* = 0.108). For Gemini (Google; Gemini 2.0), the improvement from zero-shot to few-shot was substantial, with accuracy rising from 0.500 to 0.825, macro F1 score from 0.333 to 0.824, and kappa from 0.000 to 0.650. These differences were statistically significant **(p = 0.001)**. In contrast, Copilot (Microsoft; Copilot based on GPT-5 architecture) showed a decrease in performance with few-shot prompting, as accuracy dropped from 0.775 to 0.575, macro F1 score from 0.763 to 0.379, and kappa from 0.550 to 0.171 (*p* = 0.056).

**Table 2:** Diagnostic performance of AI models limited to high-suspicion cases (VI-RADS 4–5)

	ChatGPT (OpenAI; GPT-5.2) zero shot	ChatGPT (OpenAI; GPT-5.2) few-shot	Gemini (Google; Gemini 2.0) zero shot	Gemini (Google; Gemini 2.0) few shot	Copilot (Microsoft; Copilot based on GPT-5 architecture) zero shot	Copilot (Microsoft; Copilot based on GPT-5 architecture) few shot
Accuracy	0.70 (0.53–0.83)	0.85 (0.70–0.94)	0.50 (0.34–0.66)	0.82 (0.67–0.93)	0.77 (0.61–0.89)	0.57 (0.41–0.73)
Number of Correctly Classified Reports (n)	28	34	20	33	31	23
Macro F1 score	0.69 (0.52–0.82)	0.84 (0.70–0.93)	0.33 (0.21–0.48)	0.82 (0.67–0.92)	0.76 (0.60–0.88)	0.37 (0.25–0.52)
Kappa	0.40 (0.21–0.59)	0.70 (0.51–0.89)	0.00 (0.00–0.12)	0.65 (0.46–0.84)	0.55 (0.35–0.75)	0.17 (0.05–0.31)

In the zero-shot setting, no statistically significant differences were observed between ChatGPT (OpenAI; GPT-5.2) and Copilot (Microsoft; Copilot based on GPT-5 architecture) (*p* = 0.445), ChatGPT (OpenAI; GPT-5.2) and Gemini (Google; Gemini 2.0) (*p* = 0.068), or Copilot (Microsoft; Copilot based on GPT-5 architecture) and Gemini (Google; Gemini 2.0) (*p* = 0.105). However, in the few-shot setting, ChatGPT (OpenAI; GPT-5.2) outperformed Copilot (Microsoft; Copilot based on GPT-5 architecture) significantly **(p = 0.001)**, while the performance difference between ChatGPT (OpenAI; GPT-5.2) and Gemini (Google; Gemini 2.0) was not statistically significant (*p* = 0.761). Gemini (Google; Gemini 2.0) outperformed Copilot (Microsoft; Copilot based on GPT-5 architecture) in this setting as well **(p = 0.015)**. These results suggest that in high-suspicion cases, ChatGPT (OpenAI; GPT-5.2) and Gemini (Google; Gemini 2.0) benefit from few-shot prompting, while Copilot (Microsoft; Copilot based on GPT-5 architecture) may be adversely affected.

## Discussion

The present study demonstrates the capacity of LLMs to classify bladder cancer lesions based on the VI-RADS system using synthetic MRI-based radiological scenarios. Our findings show that few-shot prompting substantially improves model performance for ChatGPT (OpenAI; GPT-5.2) and Gemini (Google; Gemini 2.0), while Copilot (Microsoft; Copilot based on GPT-5 architecture)’s performance paradoxically declined under the same condition. These results support the hypothesis that tailored prompt engineering can bridge the gap between generic language understanding and domain-specific radiological reasoning.

In recent years, artificial intelligence technologies, particularly LLMs, have increasingly attracted attention in the field of radiology as potential tools for clinical decision support and structured report interpretation. While most AI applications in radiology have traditionally focused on direct image analysis using convolutional neural networks or other computer vision techniques, LLMs offer a complementary capability by enabling the interpretation of textual radiology reports and structured clinical information. This capability may be particularly relevant in scenarios where radiological findings are summarized in narrative reports that guide downstream clinical decision-making. In this context, evaluating the ability of LLMs to interpret radiological descriptions and perform structured classification tasks such as VI-RADS scoring represents an emerging research direction that may complement image-based artificial intelligence systems [9, 10].

Inter-observer variability remains a well-recognized limitation in VI-RADS-based mpMRI interpretation, particularly in VI-RADS 3 cases, with previous studies reporting moderate inter-reader agreement among radiologists [11, 12]. This variability poses challenges in clinical decision-making, especially in distinguishing non-muscle-invasive from muscle-invasive bladder cancer. In this context, AI-assisted systems, including LLMs, have been proposed to reduce subjectivity and enhance consistency in classification. Notably, in our study, all three evaluated LLMs achieved perfect accuracy in classifying VI-RADS 3 lesions under few-shot learning conditions, suggesting that, when properly prompted, these models may offer a robust solution to one of the most ambiguous categories in bladder cancer staging. This finding distinguishes our work from prior studies and underscores the potential utility of LLMs in supporting standardized interpretation of intermediate-risk lesions.

Our observations align with recent work by Lee et al., who evaluated the performance of LLMs such as GPT-4 and Gemini in PI-RADS classification from prostate MRI reports [13]. Their study found that GPT-4 reached 83% accuracy with a macro F1 score of 0.76, and Gemini achieved 79% accuracy (F1 = 0.68), highlighting that few-shot prompting significantly enhances diagnostic precision. Similarly, our study showed marked improvements in ChatGPT (OpenAI; GPT-5.2) and Gemini (Google; Gemini 2.0) following few-shot learning (macro F1 score increased from 0.575 to 0.939 and from 0.257 to 0.823, respectively). A recent study by Çamur et al. (Korean Journal of Radiology, 2024) also investigated the ability of LLMs to interpret radiological findings within structured diagnostic frameworks. Their results similarly highlighted the potential of LLMs to support standardized interpretation of radiological reports while emphasizing the importance of appropriate prompting strategies and clinical context when applying these systems to diagnostic classification tasks [14].

In high-risk lesions (VI-RADS 4–5), both ChatGPT and Gemini showed reliable classification, which is clinically relevant as accurate staging determines the necessity for radical cystectomy or bladder-sparing approaches [15]. The relative underperformance of Copilot (Microsoft; Copilot based on GPT-5 architecture) in this subgroup further highlights the heterogeneity in LLM capabilities and the need for validation before clinical deployment.

As in other medical classification tasks, intermediate categories such as VI-RADS 3 continue to pose significant challenges for LLMs. This is consistent with findings from Lee et al. [13], who reported that PI-RADS 3 lesions were particularly difficult for both radiologists and LLMs to classify, reflecting a broader issue of diagnostic ambiguity in intermediate-risk categories. These borderline cases may benefit from hybrid approaches incorporating structured data, probabilistic modeling, or reinforcement learning techniques [16, 17].

While our study used text-only data, real-world radiology reporting involves multi-modal interpretation. Future studies should evaluate whether combining imaging features with natural language inputs improves LLM accuracy, especially for nuanced classifications. Multi-modal architectures integrating visual transformers or CNNs with LLMs have shown promise in recent AI applications [18, 19]. Explainability remains a concern in AI implementation. Despite strong accuracy metrics, current LLMs lack transparency in decision-making processes. Enhancing interpretability with attention heatmaps, probability scores, or natural language justifications will be essential for clinical adoption [20, 21].

Ultimately, institutional integration of LLMs may benefit from continuous model refinement based on feedback, structured prompting strategies, and regulatory oversight to mitigate risk in high-stakes environments like oncology and radiology.

## Limitations

This study has several limitations. First, the dataset consisted of synthetically generated radiology reports. Although these reports were constructed using expert-defined radiological descriptors and reviewed by experienced uroradiologists to ensure clinical plausibility, synthetic reports cannot fully reproduce the linguistic variability, reporting heterogeneity, and incidental noise present in real-world clinical radiology reports. Consequently, the standardized nature of the dataset may have created a more controlled evaluation environment than routine clinical practice, and the observed model performance may represent an idealized estimate.

Second, the study evaluated VI-RADS classification using text-based radiology reports rather than the original multiparametric MRI images. Because VI-RADS scoring in clinical practice relies primarily on visual interpretation of mpMRI sequences, this approach assesses the ability of LLMs to interpret structured radiological descriptions rather than their capacity to directly analyze imaging data. Therefore, the reported performance should not be interpreted as reflecting end-to-end image-based diagnostic workflows. Future studies should investigate multimodal approaches integrating both imaging data and textual reports.

Third, only three LLMs were evaluated, and the rapidly evolving nature of these systems means that model updates may influence future performance. In addition, the analyses were conducted using a single internally generated dataset without external validation, limiting the generalizability of the findings.

Another methodological consideration is that model outputs were obtained using a single-pass inference approach through publicly available web interfaces with default configurations. As a result, output variability related to stochastic decoding parameters could not be systematically evaluated. Future studies may explore repeated inference and response aggregation strategies to improve classification stability.

The repeated use of pairwise McNemar tests may also introduce limitations related to multiple comparisons, although Bonferroni correction was applied. Furthermore, retrieval-augmented generation (RAG) approaches were not evaluated and may represent a potential avenue for improving the reliability of LLM-based radiological classification. Finally, LLM outputs are known to be sensitive to prompt design and contextual framing, including the wording of instructions, the ordering of few-shot examples, and contextual window constraints. Although identical prompts were used across models, these factors may introduce variability in performance estimates.

## Conclusion

This study demonstrates that LLMs, particularly ChatGPT (OpenAI; GPT-5.2) and Gemini (Google; Gemini 2.0), can accurately classify bladder cancer lesions according to the VI-RADS system when provided with appropriate prompting. Few-shot learning significantly improved diagnostic performance for these models, highlighting the importance of contextual learning in medical AI applications. Despite variability across models and difficulties with borderline categories, LLMs show potential as decision-support tools in radiology. Future research should focus on multi-modal integration, real-world clinical validation, and strategies to improve explainability to enable safe and effective implementation in urologic oncology practice.

## Electronic supplementary material

Below is the link to the electronic supplementary material.

Supplementary Material 1

Supplementary Material 2

## Data Availability

The datasets used and/or analysed during the current study are available from the corresponding author on reasonable request.

## References

[1] MacVicar D, Husband JE. Radiology in the staging of bladder cancer. Br J Hosp Med. 1994;51(9):454–58. 7921502

[2] Gungor H, Camtosun A, Topcu I, Karaca L. Evaluating the concordance between vesical imaging reporting and data system scores and bladder tumor histopathology. Asian J Urology. 2025;12(1):87–92. 10.1016/j.ajur.2024.06.001. 10.1016/j.ajur.2024.06.001 PMC11840306 39990069

[3] Kulkarni GS, Hakenberg OW, Gschwend JE, Thalmann G, Kassouf W, Kamat A, et al. An updated critical analysis of the treatment strategy for newly diagnosed high-grade T1 (previously T1G3) bladder cancer. Eur Urology. 2010;57(1):60–70. 10.1016/j.eururo.2009.08.024 19740595

[4] Caglic I, Panebianco V, Vargas HA, Bura V, Woo S, Pecoraro M, et al. MRI of bladder cancer: local and nodal staging. J Magnetic Reson Imag. 2020;52(3):649–67. 10.1002/jmri.27090 32112505

[5] Akin O, Lema-Dopico A, Paudyal R, Konar AS, Chenevert TL, Malyarenko D, et al. Multiparametric MRI in era of artificial intelligence for bladder cancer therapies. Cancers. 2023;15(22):5468. 10.3390/cancers15225468. 38001728 10.3390/cancers15225468 PMC10670574

[6] Panebianco V, Narumi Y, Altun E, Bochner BH, Efstathiou JA, Hafeez S, et al. Multiparametric magnetic resonance imaging for bladder cancer: development of VI-RADS (Vesical imaging-reporting and data system). Eur Urology. 2018;74(3):294–306. 10.1016/j.eururo.2018.04.029 PMC6690492 29755006

[7] Rajpurkar P, et al. CheXNet: radiologist-level pneumonia detection on chest X-Rays with deep learning. 2017. arXiv: 1711.05225.

[8] Heller MT, et al. AI in radiology: impact and applications. Radiographics. 2020;40(5):1294–306.

[9] Topol EJ. High-performance medicine: the convergence of human and artificial intelligence. Nat Med. 2019;25:44–56. 30617339 10.1038/s41591-018-0300-7

[10] Jeblick K, Schachtner B, Dexl J, et al. ChatGPT makes medicine easy to swallow: an exploratory case study on simplified radiology reports. Eur Radiol. 2023;33:8099–104. 10.1007/s00330-023-10213-1 PMC11126432 37794249

[11] Caglic I, Panebianco V, Vargas HA, et al. MRI of bladder cancer: local and nodal staging. J Magn Reson Imag. 2020;52(3):649–67. 10.1002/jmri.27090 32112505

[12] Panebianco V, Narumi Y, Altun E, et al. Multiparametric magnetic resonance imaging for bladder cancer: development of VI-RADS. Eur Urol. 2018;74(3):294–306. 29755006 10.1016/j.eururo.2018.04.029 PMC6690492

[13] Lee KL, Kessler DA, Caglic I, Kuo YH, Shaida N, Barrett T. Assessing the performance of ChatGPT and Bard/Gemini against radiologists for prostate imaging-Reporting and data System classification based on prostate multiparametric MRI text reports. The Br J Radiol. 2025;98, (1167): 368–74. 10.1093/bjr/tqae236. 39535870 10.1093/bjr/tqae236 PMC11840166

[14] Çamur E, Cesur T, Güneş YC. Comparative evaluation of the accuracies of large language models in answering VI-RADS-related questions. Korean J Radiol. 2024;25(8):767–68. 10.3348/kjr.2024.0438. 39028015 10.3348/kjr.2024.0438 PMC11306009

[15] Kulkarni GS, Hakenberg OW, Gschwend JE, et al. An updated critical analysis of the treatment strategy for newly diagnosed high-grade T1 bladder cancer. Eur Urol. 2010;57(1):60–70. 19740595 10.1016/j.eururo.2009.08.024

[16] Giger ML. Machine learning in medical imaging. J Am Coll Radiol. 2018;15(3):512–20. 29398494 10.1016/j.jacr.2017.12.028

[17] Shah P, Kendall F, Khozin S, et al. Artificial intelligence and machine learning in clinical development: a translational perspective. NPJ Digit Med. 2019;2:69. 31372505 10.1038/s41746-019-0148-3 PMC6659652

[18] Rajpurkar P, Irvin J, Zhu K, et al. CheXNet: radiologist-level pneumonia detection on chest X-rays with deep learning. arXiv preprint. 2017, arXiv: 1711.05225.

[19] Esteva A, Chou K, Yeung S, et al. Deep learning-enabled medical computer vision. NPJ Digit Med. 2021;4:5. 33420381 10.1038/s41746-020-00376-2 PMC7794558

[20] Samek W, Wiegand T, Müller KR. Explainable artificial intelligence: understanding, visualizing and interpreting deep learning models. ITU J ICT Discov. 2017;1(1):39–48.

[21] Doshi-Velez F, Kim B. Towards a rigorous science of interpretable machine learning. arXiv preprint. 2017, arXiv: 1702.08608.

