# Supplementary material for: Evaluation of large language models for VI-RADS reports: a comparative analysis of zero-shot and few-shot prompting
Source: BMC Med Imaging. 2026 Apr 11;26:265. doi: 10.1186/s12880-026-02334-y (PMC13191856; doi:10.1186/s12880-026-02334-y)
Supplement: Supplementary file 1 — Supplementary Material 1 [file 12880_2026_2334_MOESM1_ESM.docx]

**Supplementary Dataset S1 – Synthetic Bladder MRI Radiology Reports**

This dataset contains 100 synthetically generated bladder MRI radiology reports used in this study. Each case includes a radiology report text and the corresponding VI‑RADS reference score (1–5).

| 1- A lesion measuring 25x18 mm located on the right inferoposterolateral bladder wall, covering the right ureteric orifice, extends into the muscular layer. Diffusion-weighted imaging shows diffusion restriction in both the tumor and muscularis propria. VI-RADS 4 |
| --- |
| 2- A 26x15 mm lesion protruding into the lumen is seen on the bladder sidewall. It shows marked diffusion restriction and early arterial enhancement, but early enhancement in the muscularis propria is uncertain. VI-RADS: 3. |
| 3- A lesion measuring approximately 48x43x25 mm is located on the posterior bladder wall, covering an area adjacent to the left ureteric orifice, with invasion into the muscular layer and perivesical fat. Diffusion restriction and early arterial enhancement are seen in the bladder wall and extravesical tissues. VI-RADS 5 |
| 4- A 7x4 mm lesion is observed on the right lateral bladder wall. It restricts diffusion and shows early enhancement without muscularis propria invasion. VI-RADS: 1. |
| 5- A lesion measuring approximately 55x50x28 mm is observed in the right lateral bladder wall, covering the right ureteric orifice, with invasion into the muscular layer and perivesical fat. Diffusion restriction and early arterial enhancement are present in the bladder wall and extravesical tissues. VI-RADS 5 |
| 6- A lesion involving a 6 cm segment of the right lateral bladder wall with a thickness of 26 mm shows extension into the extravesical area. Diffusion restriction and early arterial enhancement are seen in the bladder wall and extravesical tissues. VI-RADS 5 |
| 7- A lesion measuring 66x35 mm in its widest transverse diameter extends from the bladder base, covering the urethral orifice and along the anterior and left lateral bladder walls up to the dome. The lesion extends into the muscular layer. Diffusion-weighted imaging and dynamic contrast-enhanced sequences demonstrate diffusion restriction and early enhancement in both the tumor and muscularis propria. VI-RADS 4 |
| 8- A 24x14 mm lesion protruding into the lumen is seen on the posterior wall. It shows marked diffusion restriction and enhancement in the tumor, but early enhancement in the muscularis propria is indeterminate. VI-RADS: 3. |
| 9- A 5x5 mm lesion is located on the superior-posterior right lateral bladder wall. It restricts diffusion and shows early enhancement, but the muscularis propria is not affected. VI-RADS: 1. |
| 10- A lesion is seen at and just inferior to the left ureteral orifice, extending laterally for approximately 3 cm. It contains patchy areas of diffusion restriction, the largest measuring 4 mm. The lesion restricts diffusion and shows early arterial enhancement, but no diffusion restriction or early enhancement is seen in the muscularis propria. VI-RADS: 1. |
| 11- A 22x16 mm lesion protruding into the bladder lumen is seen on the posterior wall. It does not restrict diffusion and shows mild arterial enhancement in the tumor, with no clear findings in the muscularis propria. VI-RADS: 2. |
| 12- A diverticulum formation measuring 25 mm is observed on the anterior bladder wall. A lesion protruding into the diverticulum lumen from the inferior part of the diverticular neck extends into the extravesical fatty tissue. Diffusion-weighted imaging and dynamic contrast-enhanced sequences show diffusion restriction and early enhancement in the tumor and muscularis propria. VI-RADS 4 |
| 13- A 31x19 mm lesion protrudes into the lumen from the trigone. It shows moderate diffusion restriction and arterial enhancement in the inner layer, with questionable diffusion restriction in the muscularis propria. VI-RADS: 3. |
| 14- An 18x11x6 mm lesion is seen in the trigone area on the left. It restricts diffusion and enhances in the tumor and inner layer, with no muscularis propria involvement. VI-RADS: 2. |
| 15- A 25x15 mm lesion is located on the anterior bladder wall, protruding into the lumen. It shows marked diffusion restriction and enhancement in the tumor, with suspicious diffusion findings in the muscularis propria. VI-RADS: 3. |
| 16- A 7 mm lesion is noted at the level of the left ureteral orifice. It restricts diffusion and enhances early arterially, but the muscularis propria is spared with no restriction or enhancement. VI-RADS: 1. |
| 17- A 24x20 mm lesion is noted posteriorly near the right ureteral orifice. It restricts diffusion and shows arterial enhancement in the tumor and surrounding inner layer, with no muscularis propria involvement. VI-RADS: 2. |
| 18- An 8x8 mm lesion is located on the right lateral aspect of the bladder dome. It restricts diffusion and shows early enhancement, but the muscularis propria appears uninvolved. VI-RADS: 1. |
| 19- A diverticulum formation measuring 21 mm is observed on the right posterolateral bladder wall. A lesion protruding into the diverticular lumen from the superior aspect of the diverticular neck extends into extravesical fat. Diffusion restriction and early arterial enhancement are seen in the bladder wall and extravesical tissues. VI-RADS 5 |
| 20- A lesion measuring approximately 61x57x31 mm is observed in the left posterolateral bladder wall, covering the left ureteric orifice, with invasion into the muscular layer and perivesical fat. Diffusion-weighted imaging and dynamic contrast-enhanced sequences show diffusion restriction and early enhancement in the tumor and muscularis propria. VI-RADS 4 |
| 21- A 26x14 mm lesion is located at the bladder apex, protruding into the lumen. It shows moderate diffusion restriction and contrast enhancement in the inner layer, with inconclusive muscularis propria findings. VI-RADS: 3. |
| 22- A lesion measuring 15x10 mm with extravesical extension is observed on the right inferolateral bladder wall, including the right ureteric orifice. Diffusion restriction and early enhancement are seen in the tumor and muscularis propria. VI-RADS 4 |
| 23- A lesion involving a 3 cm segment of the right lateral bladder wall, with a thickness of 10 mm and showing extravesical extension on T2-weighted sequences, demonstrates diffusion restriction and early enhancement in the tumor and muscularis propria. VI-RADS 4 |
| 24- Multiple lesions are observed: 9 mm on the right lateral wall, 8 mm and 3 mm on the anterior wall, 3 mm on the right posterolateral wall, and 5 mm and 6 mm on the anterior dome. All show diffusion restriction and early enhancement but spare the muscularis propria. VI-RADS: 1. |
| 25- A 30x18 mm lesion protruding into the lumen is observed at the bladder base. It shows moderate diffusion restriction and marked arterial enhancement in the tumor and adjacent inner layer, with suspicious findings in the muscularis propria. VI-RADS: 3. |
| 26- A 17x11 mm lesion protruding into the lumen is seen on the lateral bladder wall. It does not restrict diffusion and shows minimal enhancement in the tumor, with no early enhancement in the muscularis propria. VI-RADS: 2. |
| 27- A 6x3 mm lesion is located in the bladder base, posterolateral to the left side of the urethra. It restricts diffusion and shows early enhancement, but the muscularis propria is uninvolved. VI-RADS: 1. |
| 28- A lesion completely covering the right lateral bladder wall shows invasion of the muscularis propria and extension into the extravesical space. Diffusion restriction and early arterial enhancement are observed in the bladder wall and extravesical tissues. VI-RADS 5 |
| 29- Two adjacent lesions measuring 4 mm and 5 mm are observed on the left lateral bladder wall. They show diffusion restriction and early enhancement, with no muscularis propria involvement. VI-RADS: 1. |
| 30- A lesion measuring 24x9 mm with extension into the muscular layer is observed on the right inferolateral bladder wall. Diffusion-weighted imaging and dynamic contrast-enhanced sequences show diffusion restriction and early enhancement in both the tumor and muscularis propria. VI-RADS 4 |
| 31- A lesion measuring 25x15 mm is located in the right posterolateral bladder wall near the right ureteric orifice, showing invasion of the muscular layer. Diffusion restriction and early arterial enhancement are noted in the bladder wall and extravesical tissues. VI-RADS 5 |
| 32- An 18x14 mm lesion protruding into the bladder lumen is observed at the apex. It has minimal diffusion restriction and slight inner layer enhancement, with no abnormalities in the muscularis propria. VI-RADS: 2. |
| 33- A 20x12 mm lesion protruding into the bladder lumen is noted on the lateral wall. It shows minimal diffusion restriction and mild arterial enhancement in the tumor and adjacent inner layer, without diffusion restriction or early enhancement in the muscularis propria. VI-RADS: 2. |
| 34- A polypoid lesion protruding into the lumen, located on the anterior bladder wall and measuring 3x3 cm in transverse diameter, extends into the extravesical fat. Diffusion restriction and early arterial enhancement are present in the bladder wall and extravesical tissues. VI-RADS 5 |
| 35- A 6x4 mm lesion is seen on the anterior bladder wall. It restricts diffusion and demonstrates early arterial enhancement without muscularis propria involvement. VI-RADS: 1. |
| 36- A 29x17 mm lesion protrudes into the lumen from the bladder base. It shows moderate diffusion restriction and arterial enhancement in the inner layer, with suspicious early enhancement in the muscularis propria. VI-RADS: 3. |
| 37- A protruding lesion into the lumen located on the anterior bladder wall, measuring 23x12 mm at its widest, shows extension into the adjacent fatty tissue in a limited area (~1 cm) at the dome level. Diffusion-weighted imaging and dynamic contrast-enhanced sequences show diffusion restriction and early enhancement in the tumor and muscularis propria. VI-RADS 4 |
| 38- A 21x14 mm lesion protruding into the lumen is seen on the anterior wall. It does not restrict diffusion and shows mild enhancement in the tumor, with no abnormalities in the muscularis propria. VI-RADS: 2. |
| 39- A 6 mm lesion protrudes into the bladder lumen from the right lateral wall. It restricts diffusion and shows early enhancement without muscularis propria involvement. VI-RADS: 1. |
| 40- A diverticulum formation measuring 20 mm is noted on the right posterolateral bladder wall. A lesion protruding into the diverticulum lumen from the superior part of the diverticular neck extends into the extravesical fatty tissue. Diffusion-weighted imaging and dynamic contrast-enhanced sequences show diffusion restriction and early enhancement in the tumor and muscularis propria. VI-RADS 4 |
| 41- A 19x15 mm lesion protruding into the bladder lumen is located at the base. It shows minimal diffusion restriction and early arterial enhancement in the inner layer, without muscularis propria involvement. VI-RADS: 2. |
| 42- A lesion measuring approximately 7x4 mm is seen medial to the left ureteral orifice. It shows diffusion restriction and early enhancement, without any involvement of the muscularis propria. VI-RADS: 1. |
| 43- A 22x13 mm lesion protruding into the lumen is seen at the bladder apex. It shows moderate diffusion restriction and contrast enhancement in the inner layer, with suspicious findings in the muscularis propria. VI-RADS: 3. |
| 44- A polypoid lesion extending into the lumen over a 38 mm segment, reaching up to 32 mm in size, is observed on the right posterior wall encompassing the right ureteral orifice. It restricts diffusion and shows enhancement in the tumor and adjacent inner layer, sparing the muscularis propria. VI-RADS: 2. |
| 45- A 15x10 mm lesion protruding into the bladder lumen is observed on the left lateral wall. It restricts diffusion and shows arterial enhancement in the tumor and adjacent inner layer, without any diffusion restriction or early enhancement in the muscularis propria. VI-RADS: 2. |
| 46- A lesion measuring 35x26 mm, protruding into the lumen from the anterior bladder wall, extends into adjacent fat tissue in a limited area (~1 cm) at the dome level. Diffusion restriction and early arterial enhancement are noted in the bladder wall and extravesical tissues. VI-RADS 5 |
| 47- A 17x14 mm lesion is located at the bladder base on the left, extending toward the urethral lumen. It shows diffusion restriction and arterial enhancement in the tumor and inner layer, but no abnormalities in the muscularis propria. VI-RADS: 2. |
| 48- A 25x14 mm lesion protruding into the lumen is located in the trigone. It shows moderate diffusion restriction and inner layer enhancement, with questionable diffusion restriction in the muscularis propria. VI-RADS: 3. |
| 49- A 6 mm lesion is noted on the anterior bladder dome, slightly left of midline. It restricts diffusion and demonstrates early arterial enhancement, but the muscularis propria is spared. VI-RADS: 1. |
| 50- A 15x13 mm lesion is observed in the left inferolateral bladder wall. It restricts diffusion and enhances in the tumor and nearby inner layer, with no signs of muscularis propria involvement. VI-RADS: 2. |
| 51- A 5x4 mm polypoid lesion protruding into the lumen is observed at the right ureteral orifice. It restricts diffusion and shows early arterial enhancement, but no findings suggesting muscularis propria involvement. VI-RADS: 1. |
| 52- A 5x4 mm lesion is observed on the left side of the anterior bladder wall. It restricts diffusion and shows early enhancement, with no evidence of muscularis propria involvement. VI-RADS: 1. |
| 53- A 20x14 mm lesion is located on the right lateral bladder wall. It restricts diffusion and shows arterial enhancement in the tumor and adjacent inner layer, without diffusion restriction or early enhancement in the muscularis propria. VI-RADS: 2. |
| 54- A 3.5 mm lesion is noted in the right inferolateral bladder wall. It restricts diffusion and shows early enhancement without muscularis propria invasion. VI-RADS: 1. |
| 55- A lesion measuring 58x30 mm in its widest transverse diameter is observed on the posterior bladder wall, covering the right ureteric orifice and extending along the right lateral bladder wall up to the dome, with invasion into the muscular layer. Diffusion-weighted imaging and dynamic contrast-enhanced sequences reveal diffusion restriction and early enhancement in the tumor and muscularis propria. VI-RADS 4 |
| 56- A lobulated polypoid lesion measuring approximately 7x6 mm is observed near the internal urethral orifice at the trigone level. It restricts diffusion and enhances early arterially, but no findings suggest involvement of the muscularis propria. VI-RADS: 1. |
| 57- A lesion measuring 23x9 mm at its widest, located on the left lateral wall of the bladder, extends beyond the bladder into the perivesical fat. Diffusion restriction and early arterial enhancement are observed in both the bladder wall and extravesical tissues. VI-RADS 5 |
| 58- A 16x12 mm lesion protruding into the lumen is located in the trigone. It shows minimal diffusion restriction and early enhancement in the inner layer, with no diffusion restriction in the muscularis propria. VI-RADS: 2. |
| 59- A lesion measuring 20x10 mm is located in the left posterolateral bladder wall, superior to the left ureteric orifice, appearing to invade the muscular layer. Diffusion restriction and early arterial enhancement are seen in the bladder wall and extravesical tissues. VI-RADS 5 |
| 60- A 26x16 mm lesion protruding into the lumen is located at the bladder base. It shows marked diffusion restriction and arterial enhancement in the tumor, with questionable muscularis propria involvement. VI-RADS: 3. |
| 61- A lesion measuring approximately 52x46x30 mm is located in the right posterolateral bladder wall, covering the right ureteric orifice, with invasion of the muscular layer and perivesical fat. Diffusion restriction and early arterial enhancement are seen in the bladder wall and extravesical tissues. VI-RADS 5 |
| 62- A 29x18 mm lesion protruding into the lumen is observed on the posterior wall. It shows marked diffusion restriction and arterial enhancement in the tumor, with early enhancement in the muscularis propria being uncertain. VI-RADS: 3. |
| 63- A 27x13 mm lesion is noted at the bladder apex, protruding into the lumen. It shows moderate diffusion restriction and enhancement in the inner layer, with inconclusive findings in the muscularis propria. VI-RADS: 3. |
| 64- A 28x16 mm lesion protrudes into the lumen from the bladder base. It shows marked diffusion restriction and arterial enhancement in the tumor, with suspicious muscularis propria involvement. VI-RADS: 3. |
| 65- A lesion measuring 24x12 mm is located on the left posterolateral bladder wall, superior to the left ureteric orifice, appearing to invade the muscular layer. Diffusion restriction and early enhancement are seen in both the tumor and muscularis propria. VI-RADS 4 |
| 66- A 22x12 mm lesion is observed on the bladder sidewall, protruding into the lumen. It demonstrates marked diffusion restriction and arterial enhancement in the tumor, with suspicious findings in the muscularis propria. VI-RADS: 3. |
| 67- A lesion with a polypoid appearance protruding into the lumen, located on the anterior bladder wall and measuring up to 6x5 cm in transverse diameter, shows evidence of muscle invasion. Diffusion restriction and early enhancement are noted in the tumor and muscularis propria. VI-RADS 4 |
| 68- A lesion measuring 13x5 mm extending into the muscular layer is observed on the right lateral wall of the bladder. Diffusion restriction and early enhancement are noted in the tumor and muscularis propria on both diffusion-weighted imaging and dynamic contrast-enhanced sequences. VI-RADS 4 |
| 69- A lesion measuring approximately 70x62x35 mm is located on the anterior bladder wall, extending to the trigone, with invasion of the muscular layer and perivesical fat. Diffusion restriction and early arterial enhancement are noted in the bladder wall and extravesical tissues. VI-RADS 5 |
| 70- A polypoid lesion protruding into the lumen is observed on the left inferolateral bladder wall, measuring 5x4 cm in its widest transverse diameter, with evidence of muscle invasion. Diffusion-weighted imaging and dynamic contrast-enhanced sequences show diffusion restriction and early enhancement in the tumor and muscularis propria. VI-RADS 4 |
| 71- A 28x16 mm lesion protruding into the lumen is located in the trigone. It shows moderate diffusion restriction and arterial enhancement in the inner layer, with suspicious diffusion findings in the muscularis propria. VI-RADS: 3. |
| 72- A 24x14 mm lesion protruding into the lumen is seen on the anterior bladder wall demonstrates marked diffusion restriction and arterial enhancement in the tumor, with suspicious findings in the muscularis propria. VI-RADS: 3. |
| 73- A 23x13 mm lesion is seen on the posterior wall, protruding into the lumen. It shows marked diffusion restriction and enhancement in the tumor, but early enhancement in the muscularis propria is uncertain. VI-RADS: 3. |
| 74- A lesion measuring approximately 61x57x31 mm in the left posterolateral bladder wall, covering the left ureteric orifice, extends into the muscular layer and perivesical fat. Diffusion restriction and early arterial enhancement are seen in the bladder wall and extravesical tissues. VI-RADS 5 |
| 75- A 30x17 mm lesion protruding into the lumen is observed on the bladder sidewall. It shows moderate diffusion restriction and enhancement in the inner layer, with suspicious early enhancement in the muscularis propria. VI-RADS: 3. |
| 76- A diverticulum formation measuring 18 mm is seen on the left posterolateral bladder wall. A lesion protruding into the diverticulum lumen from the lateral part of the diverticular neck extends into the extravesical fatty tissue. Diffusion-weighted imaging and dynamic contrast-enhanced sequences show diffusion restriction and early enhancement in the tumor and muscularis propria. VI-RADS 4 |
| 77- A 25x13 mm lesion protruding into the lumen is located in the trigone. It has minimal diffusion restriction and shows early enhancement in the inner layer, with no restriction in the muscularis propria. VI-RADS: 2. |
| 78- A lesion measuring 18x10 mm is located in the trigone on the right side, with invasion into the muscular layer. Diffusion restriction and early arterial enhancement are seen in the bladder wall and extravesical tissues. VI-RADS 5 |
| 79- A lesion measuring 16x8 mm protruding into the lumen is observed on the left lateral wall of the bladder, with evidence of muscularis propria invasion. Diffusion-weighted imaging and dynamic contrast-enhanced sequences show diffusion restriction and early enhancement in both the tumor and the muscularis propria. VI-RADS 4 |
|  |
| 80- A lesion measuring 34x24 mm is located in the right inferolateral bladder wall, involving the right ureteric orifice, and shows extravesical spread. Diffusion restriction and early arterial enhancement are noted in the bladder wall and extravesical tissues. VI-RADS 5 |
| 81- A 23x17 mm lesion protruding into the lumen is seen on the posterior wall. It does not restrict diffusion and shows minimal arterial enhancement in the tumor, with no enhancement in the muscularis propria. VI-RADS: 2. |
| 82- A 15x10 mm lesion protruding into the bladder lumen is seen at the base. It does not restrict diffusion and shows weak arterial enhancement in the tumor, with no signs of muscularis propria involvement. VI-RADS: 2. |
| 83- Two lesions, the largest measuring 4 mm, are observed just inferior to the right ureteral orifice. They restrict diffusion and show early arterial enhancement, but no diffusion restriction or early enhancement is seen in the muscularis propria. VI-RADS: 1. |
| 84- A 27x17 mm lesion is seen on the anterior wall, protruding into the lumen. It demonstrates marked diffusion restriction and arterial enhancement in the tumor, with suspicious muscularis propria findings. VI-RADS: 3. |
| 85- A 15x8 mm lesion is located at the junction of the right lateral bladder wall and base. It restricts diffusion and shows arterial enhancement in the tumor and adjacent inner layer, without involvement of the muscularis propria. VI-RADS: 2. |
| 86- An 8 mm exophytic lesion is seen at the level of the right ureteral orifice. It restricts diffusion and shows early arterial enhancement, but no diffusion restriction or early enhancement is seen in the muscularis propria. VI-RADS: 1. |
| 87- A 16x15 mm lesion is seen on the antero-inferior bladder wall. It restricts diffusion and shows arterial enhancement in the tumor and neighboring inner layer, sparing the muscularis propria. VI-RADS: 2. |
| 88- A lesion starting from the right posterolateral bladder wall, covering the right ureteric orifice and extending posteriorly to the bladder dome, measures 67x20 mm at its widest and extends into the perivesical fat. Diffusion restriction and early arterial enhancement are observed in the bladder wall and extravesical tissues. VI-RADS 5 |
| 89- A lesion measuring 13x12 mm in the right inferolateral bladder wall, involving the right ureteric orifice, appears to invade the muscle layer. Diffusion restriction and early arterial enhancement are noted in the bladder wall and extravesical tissues. VI-RADS 5 |
| 90- A lesion measuring approximately 45x38x22 mm is observed on the right lateral bladder wall near the right ureteric orifice, with extension into the muscular layer and perivesical fatty tissue. Diffusion restriction and early enhancement are noted in the tumor and muscularis propria on diffusion-weighted imaging and dynamic contrast-enhanced sequences. VI-RADS 4 |
| 91- A lesion measuring 48x39x43 mm located on the posterior bladder wall shows extension into the extravesical space. Diffusion restriction and early arterial enhancement are present in the bladder wall and extravesical tissues. VI-RADS 5 |
| 92- A lesion measuring 55x30 mm at its widest, located on the anterior bladder wall, demonstrates extension into extravesical fat. Diffusion restriction and early arterial enhancement are present in the bladder wall and extravesical tissues. VI-RADS 5 |
| 93- A 22x18 mm lesion protruding into the bladder lumen is seen on the posterior wall at the level of the ureteral orifice. It restricts diffusion and shows arterial enhancement in the tumor and adjacent inner layer, but the muscularis propria is spared. VI-RADS: 2. |
| 94- A lesion measuring 25x13 mm adjacent to the right ureteric orifice, with evidence of muscularis propria invasion, demonstrates early enhancement in both the tumor and muscularis propria on dynamic contrast-enhanced sequences. VI-RADS 4 |
| 95- A lesion measuring approximately 40x30 mm is seen in the left posterolateral bladder wall, covering the left ureteric orifice, with invasion of the muscular layer and perivesical fat. Diffusion-weighted imaging and dynamic contrast-enhanced sequences show diffusion restriction and early enhancement in the tumor and muscularis propria. VI-RADS 4 |
| 96- A 4x3 mm lesion is seen near the right ureteral orifice. It restricts diffusion and enhances early arterially, without muscularis propria involvement. VI-RADS: 1. |
| 97- A lesion measuring 28x14 mm is located at the bladder dome, showing invasion of the muscular layer. Diffusion restriction and early arterial enhancement are noted in the bladder wall and extravesical tissues. VI-RADS 5 |
| 98- A 24x15 mm lesion protrudes into the lumen from the bladder sidewall. It shows moderate diffusion restriction and enhancement in the inner layer, with suspicious early enhancement in the muscularis propria. VI-RADS: 3.  99- A 4 mm lesion is observed in the inferolateral portion of the bladder adjacent to the left anterolateral urethra. The lesion restricts diffusion and shows early arterial enhancement, but no diffusion restriction or early enhancement is detected in the muscularis propria. VI-RADS: 1. |

100- A lesion measuring 52x42 mm with muscular layer invasion is located at the anterior bladder dome. Dynamic contrast-enhanced sequences reveal early enhancement in the tumor and muscularis propria. VI-RADS 4
