# Supplementary material for: Evaluation of large language models for VI-RADS reports: a comparative analysis of zero-shot and few-shot prompting
Source: BMC Med Imaging. 2026 Apr 11;26:265. doi: 10.1186/s12880-026-02334-y (PMC13191856; doi:10.1186/s12880-026-02334-y)
Supplement: Supplementary file 2 — Supplementary Material 2 [file 12880_2026_2334_MOESM2_ESM.docx]

# TRIPOD‑LLM Checklist

Manuscript: Evaluation of Large Language Models for VI‑RADS Reports: A Comparative Analysis of Zero‑Shot and Few‑Shot Prompting

| Section | Item | TRIPOD‑LLM Recommendation | Reported in Manuscript |
| --- | --- | --- | --- |
| Title | Identify the study as an AI/LLM evaluation | Title should indicate AI/LLM evaluation | Title page |
| Abstract | Structured abstract | Summary of objective, methods, models, and results | Abstract |
| Introduction | Background and rationale | Explain clinical context and motivation | Introduction |
| Introduction | Study objective | State the aim of evaluating LLM performance | Introduction (last paragraph) |
| Methods | Data source | Describe dataset used | Materials and Methods |
| Methods | Case design | Explain synthetic dataset design | Materials and Methods |
| Methods | Outcome definition | Define VI‑RADS classification outcome | VI‑RADS assessment section |
| Methods | Predictors/input | Describe radiological descriptors used | Materials and Methods |
| Methods | Model description | Specify LLMs evaluated | Materials and Methods |
| Methods | Prompt strategy | Explain zero‑shot and few‑shot prompting | Reported |
| Methods | Exact prompts | Provide exact prompts used | Reported in Supplementary Material |
| Methods | Model version details | Specify model versions | Partially reported |
| Methods | Model access details | Platform and access date | Not reported |
| Methods | Inference parameters | Temperature / sampling parameters | Not reported |
| Methods | Evaluation metrics | Accuracy, F1 score, kappa | Statistical analysis |
| Methods | Statistical testing | Describe statistical tests | Statistical analysis |
| Results | Model performance | Report results | Results |
| Discussion | Interpretation | Clinical interpretation of results | Discussion |
| Discussion | Limitations | State study limitations | Limitations section |
| Other | Funding | Declare funding sources | Declarations |
| Other | Conflict of interest | Declare conflicts | Declarations |
